# Structural characteristic and phylogenetic analysis of the complete chloroplast genome of *Dianthus Caryophyllus*

**DOI:** 10.1080/23802359.2018.1521313

**Published:** 2018-10-08

**Authors:** Shuwen Chen, Zhenggang Xu, Yunlin Zhao, Xiaofen Zhong, Chaoyang Li, Guiyan Yang

**Affiliations:** a Key Laboratory of Economic Plant Resources Development and Utilization in Shaanxi Province, College of Forestry, Northwest A & F University, Yangling, Shaanxi, China;; b Hunan Research Center of Engineering Technology for Utilization of Environmental and Resources Plant, Central South University of Forestry and Technology, Changsha, Hunan, China;; c School of Material and Chemical Engineering, Hunan City University, Yiyang, Hunan, China

**Keywords:** *Dianthus caryophyllus*, structural characteristic, phylogenetic analysis, chloroplast genome

## Abstract

Chloroplast genomes are widely used in genetic engineering, molecular marker development, and phylogeny. In order to analyze the complete chloroplast genome of *Dianthus caryophyllus*, the complete chloroplast genome of *D. caryophyllus* was sequenced and annotated. On the other hand, phylogenetic analysis of the chloroplast genome of *D. caryophyllus* was carried out. The results showed that the whole length of the chloroplast genome of *D. caryophyllus* was 147,604 bp, and had a typical conserved quadripartite structure. The G and C basic content of *D. caryophyllus* chloroplast was 36.3%. The genome contained 83 protein-coding genes, 34 tRNA genes, and 6 rRNA genes. Among the protein-coding genes, 10 genes contain a single intron and 2 genes contain two introns. The phylogeny of *D. caryophyllu*s indicated that the closest phylogenetic relationship was *D. longicalyxanus*. This study provides materials for the molecular study of *D. caryophyllus* may improve the carnation industry.

*Dianthus caryophyllus*, the carnation or clove pink, belong to Caryophyllaceae and is an important floriculture. The plant probably native to the Mediterranean region but now extend widely during the cultivation process (Huxley 1992). At the same time, the genetic background is unclear particularly to garden hybrids between *D. caryophyllus* and other species in the genus. Recent study main focused on the nucleus genetic (Kong et al. 2018). Enhanced by economic value, it is necessary to explore the characteristics of complete chloroplast genome and analyze the phylogenetic position for *D. caryophyllus*.

The *D. caryophyllus* cultivar was planted in the experiment yard of Central South University of Forestry and Technology (Changsha, China). Young leaves for research were collected before blooming and stored in −80 °C refrigerator with accession QA0511. Total genomic DNA was extracted using a QIAquick DNA Mini Kit (QIAGEN, Valencia, CA, USA) according to the manufacturer’s instructions. Shotgun libraries were constructed and sequencing was sequenced on the Illumina HiSeq 2500 plantform (Illumina, CA, USA). Chloroplast genome data were filtered, annotated, and the circular map was generated as described by Zhang et al. (2017). The sequence was submitted to NCBI dataset with accession number MG989277. Then 21 close relationship species were selected to analyze the genetic relationship between them and *D. caryophyllus.* The analysis involved all the chloroplast genome of the above species and the evolutionary analyses were conducted in iTOL software (Heidelberg, Germany) using Neighbor-Joining method (Letunic and Bork 2016).

The circular molecule length of *D. caryophyllus* chloroplast genome is 147,604 bp in length, with a large single copy (LSC) region of 84,775 bp, a small single copy (SSC) region of 17,102 bp, and a pair of inverted repeats (IRs) regions of 22,863 bp. The total G + C content is 36.30%, and it is 42.80% of IRs, which was higher than the LSC and SSC regions (34.10 and 29.80%, respectively). One hundred and twenty-three genes were annotated in the genome, including 83 protein-coding genes, 34 tRNA genes, and 6 rRNA genes, of which 12 were duplicated in the IRs region. Among the protein-coding genes, 10 genes (*rps16*, *atpF*, *rpoC1*, *petB*, *petD*, *rpl16*, *ndhB*, *ndhA*, *ycf1*, and *ndhB*) contain a single intron and 2 genes (*ycf3* and *clpP*) contain two introns. The phylogenetic analysis indicated that there was closest relationship between *D. caryophyllus* and *D. longicalyx* (Figure 1). Basing on the analysis, Caryophyllales was clustered into two group, clade one with Caryophyllaceae and Amaranthaceae, clade two with Montiaceae, Talinaceae, Portulacaceae, and Aizoaceae. Chloroplast genomes are widely used in genetic engineering, molecular marker development, and phylogeny (Ivanova et al. 2017). The chloroplast genome of *D. caryophyllus* laid a good foundation for genetic resources conservation and may improve the carnation industry.

**Figure 1.**
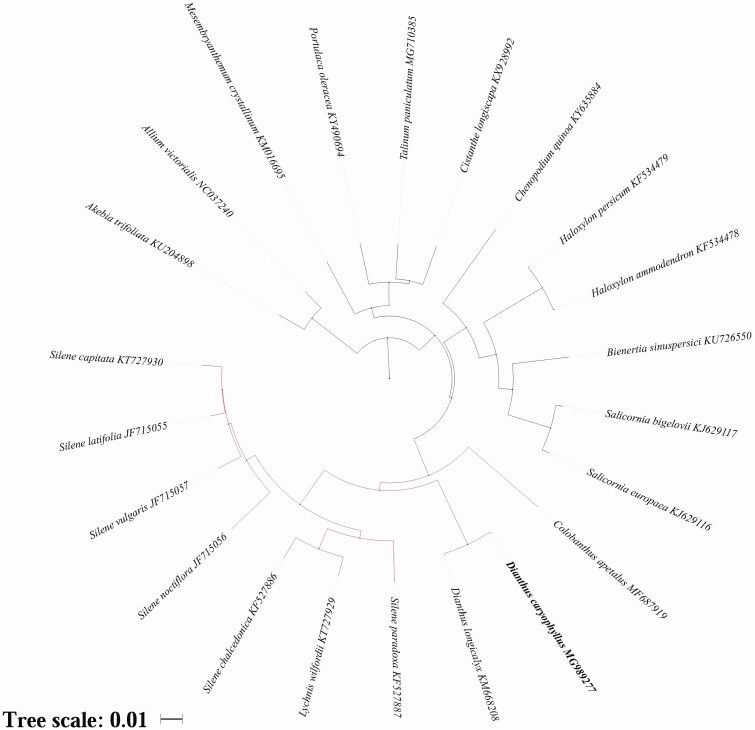
Phylogenetic analysis involved all the chloroplast genome of 2 species using neighbor-joining (NJ) analysis in iTOL.
